# Human Stem Cell Transplantation for Retinal Degenerative Diseases: Where Are We Now?

**DOI:** 10.3390/medicina58010102

**Published:** 2022-01-10

**Authors:** Ignacio Alcalde, Cristina Sánchez-Fernández, Carla Martín, Nagore De Pablo, Nahla Jemni-Damer, Gustavo V. Guinea, Jesús Merayo-Lloves, Susana Del Olmo-Aguado

**Affiliations:** 1 Instituto Universitario Fernández-Vega, Fundación de Investigación Oftalmológica, Universidad de Oviedo, 33012 Oviedo, Spain; criis.san.fer.94@gmail.com (C.S.-F.); cmartincueto@gmail.com (C.M.); nagore.depablo@fio.as (N.D.P.); merayo@fio.as (J.M.-L.); solmo@fio.as (S.D.O.-A.); 2 Instituto de Investigación Sanitaria del Principado de Asturias (ISPA), 33011 Oviedo, Spain; 3 Department of Functional Biology, University of Oviedo, 33006 Oviedo, Spain; 4 Neuro-Computing and Neuro-Robotics Research Group, Complutense University of Madrid, 28040 Madrid, Spain; nahlajem@ucm.es; 5 Innovation Group, Institute for Health Research San Carlos Clinical Hospital (IdISSC), 28040 Madrid, Spain; 6 Center for Biomedical Technology (CTB), Universidad Politécnica de Madrid, 28223 Madrid, Spain; gustavovictor.guinea@ctb.upm.es; 7 Departamento de Ciencia de Materiales, ETSI Caminos, Canales y Puertos, Universidad Politécnica de Madrid, 28040 Madrid, Spain; 8 Biomedical Research Networking Center in Bioengineering, Biomaterials and Nanomedicine (CIBER-BBN). 28040 Madrid, Spain; 9 Biomaterials and Regenerative Medicine Group, Instituto de Investigación Sanitaria del Hospital Clínico San Carlos (IdISSC), 28040 Madrid, Spain

**Keywords:** retina, stem cells, transplantation, clinical trial

## Abstract

*Background and Objectives*: Irreversible visual impairment is mainly caused by retinal degenerative diseases such as age-related macular degeneration and retinitis pigmentosa. Stem cell research has experienced rapid progress in recent years, and researchers and clinical ophthalmologists are trying to implement this promising technology to treat retinal degeneration. The objective of this systematic review is to analyze currently available data from clinical trials applying stem cells to treat human retinal diseases. *Materials and Methods*: We performed a systematic literature search in PubMed to identify articles related with stem cell therapies to retinal diseases published prior to September 2021. Furthermore, a systematic search in ClinicalTrials (NIH U.S. National Library of Medicine) was performed to identify clinical trials using stem cells to treat retinal diseases. A descriptive analysis of status, conditions, phases, interventions, and outcomes is presented here. *Conclusions:* To date, no available therapy based on stem cell transplantation is approved for use with patients. However, numerous clinical trials are currently finishing their initial phases and, in general, the outcomes related to implantation techniques and their long-term safety seem promising. In the next few years, we expect to see quantifiable results pertaining to visual function improvement.

## 1. Introduction

Irreversible visual impairment is mainly caused by retinal degenerative diseases such as age-related macular degeneration (AMD) and retinitis pigmentosa (RP). Macular diseases cause the loss of the retinal pigment epithelium together with the photoreceptors that support it [1]. It has been estimated that AMD had affected 196 million people by 2020 [2,3,4], while RP affects about 2.5 million people worldwide [4,5]. These numbers are projected to heavily increase in the next 20 years, posing substantial social and economic concerns [2,3].

There is currently no surgical or pharmacological solution to regenerate an injured or degenerative retina, and the only approach ophthalmologists can take is to slow the progress of the loss of vision. However, stem cell research has experienced rapid development in recent years. In the last decade, many efforts have been made to take advantage of the promising properties of the stem cell technology and apply them to retinal degenerative diseases. Pluripotent stem cells (PSCs) have the ability to undergo self-renewal and to give rise to all cells of the tissues of the body [6]. There are two types of PSCs depending on their origin, namely embryonic stem cells (ESC) and induced pluripotent stem cells (iPSCs) [7,8]. Since the refinement of methods to obtain large amounts of pluripotent cells from human adult tissues obviates the necessity for the use of embryonic tissue to isolate PSCs, there has been an increased interest in different medical applications of these cell technologies. Advances in cell processing have included the isolation of different adult somatic cells candidates to be dedifferentiated and induced to become PSCs, as well as the identification of new adult stem cells niches (and the isolation of many of them to be differentiate to other cell types). Most effort to date has been on the conversion of mesenchymal stem cells to muscle fibers, cardiomyocytes, B cells of the pancreas, or neurons.

The use of retinal pigment epithelial cells (RPE) and photoreceptor replacement have been investigated in implantation formats, such as an injection of cells or the placing of a sheet of cells over a substrate [9]. In this review, we will focus on the analysis of the currently available clinical trials data involving stem cells to treat human retinal diseases.

## 2. Methods

We performed a systematic literature search in PubMed to identify articles on stem cell therapies related to retinal diseases published up to September 2021. The search term used was “Stem cell transplant AND retina”. Articles were subsequently selected using the following PubMed filters: “Human”, “Adult: +19 years”, Child: birth–18 years”, and “Clinical Trials”. In addition, a systematic search in ClinicalTrials.gov (NIH U.S. National Library of Medicine) was carried to identify clinical trials using stem cells to treat retinal diseases. The search terms used were: Status “All studies”; Condition or disease “Retina Disorder”, “Retinal degeneration”, “Retinal dystrophies”; Other terms “Stem cells”; Country “Empty”. A descriptive analysis of status, conditions, phases, interventions and outcomes is presented below.

## 3. Retinal Therapies Using Stem Cells in the Literature Search

The first level of search using “Stem cell transplant AND retina” keywords produced 1229 publications indexed in PubMed. By using the filter “Human” we excluded 459 of the studies. Two additional filters were subsequently applied in order to ensure that the aim of the studies focused on patient intervention and excluded in vitro or animal experimental procedures. We used the “Adult: 19+ years” PubMed filter followed by “Child: birth- 18 years”, which resulted in a data set of 121 studies. We then identified 12 review articles and 12 clinical trials by activating the corresponding filters in PubMed. Two of the clinical trials were not related to retinal treatments and were excluded from the study. A summary of the conclusions of the 10 papers containing results from clinical trials and published between 2014 and 2021 [10,11,12,13,14,15,16,17,18,19] is presented in Table 1.

## 4. Clinical Trials Using Stem Cells to Treat the Human Retina

**Figure 1:**
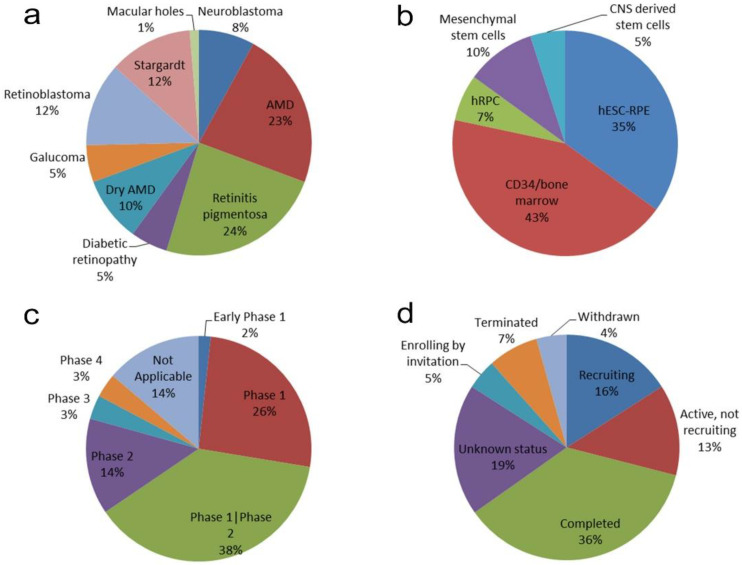
Summary of findings of the analysis of 69 Clinical trials using human stem cells for transplantation into diseased human retina. Sector graphs showing: (**a**) clinical trials with respect to the diseases involved; (**b**) the different sources of the stem cells used; (**c**) the stage of the trials at the time of the published work; (**d**) the current status of the trials.

The systematic search for clinical trials in the NIH database ClinicalTrials.gov produced 82 results using the searching terms “Retina Disorder” + “Stem cells”. The list was then manually filtered and 13 studies were excluded that did not actually use stem cells as therapy. The 69 remaining records included 31 studies that were also obtained using the search terms “Retinal dystrophies” + “Stem Cells” and 58 which were also found using “Retinal Degeneration” + “Stem Cells”. A list of the clinical trials used for this work is provided as supplementary material “Review of Clinical Trials” and their main characteristics are summarized in Figure 1.

The majority of the clinical trials studied disease conditions related to the degenerative loss of photoreceptors and/or RPE cells, such as AMD (33%), RP (24%), or Stargardt disease (12%), while the most prevalent degenerative diseases of the retina, such as glaucoma, only accounted for a 5% of the studies. Furthermore, 20% of the trials were dedicated to studying ocular tumors (Figure 1a). Thus, it can be seen that the efforts of clinicians and stem cell researchers have focused on photoreceptor and RPE replacement using alternative sources of cells.

Of the 69 clinical trials selected according to our search criteria, 9 were found not to use cells as a transplantation therapy. These include the use of optical coherence tomography (OCT) to evaluate changes in the ganglion cell complex (GCC) in diabetes (NCT02620644) or the evaluation of drugs to treat diabetic retinopathy (NCT01927315; NCT02353923). In addition, the objective of six of the trials was to obtain iPSCs for research purposes from the skin, hair or peripheral blood biopsies of patients with retinoblastoma (NCT02193724), diabetes (NCT03403699), AMD (NCT03372746; NCT02464956; NCT01432847), or other retinal diseases such as macular dystrophies (NCT02162953).

A total of 58.3% of the trials (35 out of 60) used cells from extraocular sources, with autologous bone marrow stem cells being the cell type preferred to prepare the transplantation product (in 26 cases), followed by mesenchymal stem cells of umbilical cord origin (NCT03437759; NCT04224207; NCT04315025; NCT04763369) and adipose tissue (NCT02024269; NCT02144103). Furthermore, there were three clinical trials using neuronal precursors obtained from fetal brains for retinal transplantation (Figure 1b), all of which were sponsored by StemCells, Inc. Two of these studies were terminated on the basis of a business decision unrelated to safety concerns, as reported by the company (NCT02467634; NCT02137915), while, although the other was completed, no results were posted (NCT01632527).

The use of neuronal cells to repair retinal degeneration would seem to be the most appropriate strategy due to the similarity between transplanted cells and the degenerated retinal neurons, even when 88.3% of the 60 trials were based on the use of non-neuronal cells (Figure 1b). The more suitable situation would be the use of retinal neuronal cells. One of the best-known stem cell therapies in retinal disease is “photoreceptor replacement therapy”, which can be used in various retinopathies in a way that allows the restoration of visual function [20]. Numerous preclinical studies have reported a modest degree of synaptic integration of the transplanted cells into the host retina, suggesting some capacity for regeneration [21,22,23]. In the process of the degeneration of the mammalian retina, the underlying retinal circuit remains well-preserved for a long period of time, thus allowing these circuits to be reactivated through cell therapy so that the photosensitivity of the degenerating retina could be restored [20]. In stem cell-derived rod photoreceptor precursor transplants, improvements in visual functioning were initially thought to occur since donor cells were integrated into the host’s retina, but later studies showed that what occurs after transplantation is an exchange of cytoplasmic material between the transplanted cells and the cells of the host retina, thereby enabling them to acquire the proteins they lacked [1]. The benefit of neural cell transplantation may, therefore, be driven by the neuroprotective/regenerative effects of the neurotransmitters and growth factors delivered by the transplanted cells. Four clinical trials identified by our systematic search used human retinal precursor cells (hRPCs), which were presumed to be of embryonic neuronal origin. All four used advanced cellular products from the Biotech companies JCyte (NCT04604899; NCT03073733; NCT02320812) and Reneuron (NCT02464436), understandable as hRPC treatment may be more specific in its therapeutic mechanism as compared to bone marrow stem cells or mesenchymal stem cells [9]. The problem with obtaining newborn photoreceptors, however, is that there is not an unlimited source [24]. Furthermore, neuronal cells account for additional difficulties due to their inability to be expanded in vitro as well as their low survival rate after isolation or transplantation. In the studies mentioned here, however, the researchers obtained precursor cells from human fetuses, thus increasing their survival capacity. The counterpoint to this, however, is the difficulty of obtaining the correct in situ differentiation and thus increasing the risk of tumorigenesis. This fact reduces their feasibility in transplantation procedures. At the date of this review, two of the trials had completed phase 1 and phase 2 studies, one of them with results available at ClinicalTrials.gov (https://ClinicalTrials.gov/show/NCT02320812 (accessed on 10 September 2021)). This study involved 28 participants and only one case of serious adverse effects (migratory pain) was found, while all cases reported improved visual acuity, suggesting the safety and feasibility of the procedure. The other two trials are also in phases 1 and 2, providing support for the safety of hRPC transplantation.

However, the preferred source of ocular cells is not a neural cell. Twenty-one trials used RPE cells or cells induced to give rise to RPE (Figure 1b). There is already technical evidence for the successful implantation of RPE cells and reported improvement in visual function. The cases reviewed by Jha and Bharti [25] that used a small piece of autologous RPE-choroid from the peripheral retina for implantation into macular atrophic areas in AMD patients evidenced the long-term integration of the transplant, as well as vision stabilization for several years. A phase 2 clinical trial in which human fetal neural retinal tissue and RPE were co-transplanted in AMD patients (NCT00346060), showed promising results in terms of vision improvement six months post-implantation [26].

Furthermore, RPE cells seem to be more suitable for transplantation as they do not require synaptic connections to adjacent cells [27]. Transplantation of hESC-derived RPE in AMD patients has shown good preliminary results in terms of safety and tolerability. The results from the studies using hESC-derived RPE are promising and supports the notion of exgtending this line of research in the future to randomized multicenter trials using functional measures to appropriately evaluate its efficacy [9].

## 5. Different Administration Routes for Different Types of Stem Cells

Intravitreal injection of cell suspensions is the method of choice for transplantation of bone marrow stem cells in the majority of clinical trials. Also, 3 of the studies reviewed here used this route to deliver RPC into the eye. The intravitreal injection of a suspension of cells does, however, have drawbacks such as the need for the cells to migrate from the injection site to the site of cell loss, incomplete maturation and differentiation into specific phenotypes, and the fact that they have to form connections between each other, as well as with other cells in the diseased eye, in order to remain functional.

The subretinal space is the location selected by all of the clinical trials using RPE-based strategies (21 out of 60) and also by one study using RPC. Injections of cell suspensions of human RPE and of photoreceptors alone in the subretinal space have demonstrated partial vision restoration in preclinical models of early retinal degeneration [28,29,30,31] and the applicability of these injections in patients is currently in phase 1/2 clinical trials. The transplantation of cells as a sheet partially overcomes the inconveniences of injections, as they can immobilize and support the cells. Transplanted retinal sheets have shown better survival compared to RPE injected as a suspension [32], as well as the formation of synapses with other neuronal retinal cells months after implantation (and even visual recovery, in some cases). Polymeric biomaterials such as parylene, polyester, and poly (lactide-co-glycolic acid) (PLGA) sheets have been used to seed hESC-derived RPE cells and iPSC-derived RPE cells in phase 1 and 1/2 studies of implantation in AMD patients (NCT02590692; NCT04339764; NCT01691261).

## 6. Current Status of Clinical Trials

The majority of the trials (68%) were in the initial phases of their studies (phases 1 and 2), which correspond to safety and feasibility outcomes. Only 2 of 69 trials reported reaching phase 4 and other 2 has reached phase 3 (Figure 1c). Eight studies were withdrawn or terminated prior to the scheduled end-date. Only 14 of the 69 clinical trials were currently recruiting or enrolling patients while other 9 trials were active but not recruiting patients. Only 3 trials had posted results at ClinicalTrials.gov. Although 36% of the trials were completed, only two of them had posted results. Seven articles from our PubMed search had published results linked with 8 of the clinical trials [10,11,13,14,15,16,19].

## 7. Future Directions

The problem with obtaining viable human compatible cells (photoreceptors and RPE) is that there is not an unlimited source, since so many groups have focused on obtaining this [24]. hiPSCs have been explored as a near-unlimited cell source for cell replacement of both types of retinal cells, photoreceptors and RPE cells [7,33,34,35,36,37]. Indeed, a safety and tolerability prospective clinical trial is currently underway to evaluate subretinal transplantation of iPSC-derived RPE cell sheets in AMD patients (NCT04339764). Additionally, five clinical trials are active or completed whose objective was to obtain hiPSCs for transplantation into diseased retinas, although none of the studies involve surgical procedures in patients (NCT03403699; NCT03372746; NCT02464956; NCT02162953; NCT01432847). Due to the intrinsic complexity of neural tissue reconnection, the use of neuronal precursors or in vitro differentiated neuronal constructs in retinal transplantation seems to still require further preclinical development. One interesting use of stem cells is the creation of organoids, which consist of a tiny three-dimensional mass of tissue developed in the laboratory by growing stem cells. It is possible to grow organoids that resemble human tissues or organs, or a specific type of tumor (NIH). Consequently, iPSCs can be reprogrammed to generate a retina from which an enormous amount of information about the transcriptional and epigenetic regulation of retinal development and maintenance can be obtained [38]. In addition, they serve to advance cell therapies to treat diseases such as AMD and glaucoma [38]. Besides, self-organizing optic cups and 3D neural retinas that could be clinically applied for transplantation therapy in retinal degeneration have been successfully created from hiPSCs by 3D differentiation culture [36,37,39].

## 8. Conclusions

To date there is no available therapy based on stem cell transplantation that has been approved for use with patients. Numerous clinical trials are currently finishing the early phases of their studies and, in general, the outcomes related with implantation techniques and long-term safety seem promising, including at five-year follow-up after transplantation. In the next few years, we expect to see quantifiable results in terms of visual function improvement. The use of stem cells in eye regeneration is slowly becoming positioned as an exciting retinal strategy for treating degenerative diseases. However, there is still a need for more in-depth research to increase our understanding of the mechanisms underlying stem cell differentiation into the different retinal neuronal phenotypes and to develop techniques to achieve synaptic integration and circuitry functional restoration.

## Figures and Tables

**Table 1:** Summary of the results of the articles linked to clinical trials using stem cells to treat human retinal diseases.

Title	Disease	Intervention	Results
Human embryonic stem cell (hESC)-derived retinal pigment epithelium in patients with age-related macular degeneration and Stargardt macular dystrophy: follow-up of two open-label phase 1/2 studies (NCT01345006 and NCT01344993) [14]	Stargardt macular dystrophy.	Subretinal transplantation of hESC-derived retinal pigment epithelium	Evidence of the medium-term to long-term safety, graft survival, and possible biological activity of pluripotent stem cell progeny in individuals with any disease.
	Atrophic age-related macular degeneration.		
Phase 1 clinical study of an embryonic stem cell-derived retinal pigment epithelium patch in age-related macular degeneration (NCT01691261) [10]	Severe exudative AMD.	Subretinal implant of engineered human embryonic stem cell (hESC)-derived RPE patch.	Successful delivery and survival of the RPE patch.
		data	Visual acuity gain of 29 and 21 letters.
Transplantation of Human Embryonic Stem Cell-Derived Retinal Pigment Epithelial Cells in Macular Degeneration (NCT01469832) [15]	Stargardt disease (STGD1).	Subretinal transplantation of up to 200,000 hESC-derived RPE cells with systemic immunosuppressive therapy for 13 weeks	Survival of viable transplanted hESC-derived RPE cells.
Long-term safety and tolerability of subretinal transplantation of embryonic stem cell-derived retinal pigment epithelium in Asian Stargardt disease patients (NCT01625559) [16]	Stargardt macular dystrophy (SMD).	Subretinal transplantation of hESC-retinal pigment epithelium (RPE) cells	No serious adverse events. Long-term safety, tolerability and feasibility.
			Favorable functional and anatomical results.
Surgical Method for Implantation of a Biosynthetic Retinal Pigment Epithelium Monolayer for Geographic Atrophy: Experience from a Phase 1/2a Study (NCT02590692) [11]	Advanced non-neovascular age-related macular degeneration (NNAMD).	Subretinal implantation of a human embryonic stem cell-derived retinal pigment epithelium (RPE) monolayer seeded on a synthetic substrate.	No unanticipated serious adverse events related to the implant or surgery were reported.
			Surgical implantation of CPCB-RPE1 targeted to the area of GA in subjects with advanced NNAMD is feasible in an outpatient setting.
Subretinal adipose tissue-derived mesenchymal stem cell implantation in advanced stage retinitis pigmentosa: a phase I clinical safety study (Ministry of Health of Turkey approval: 56733164/203) [17]	Retinitis pigmentosa.	Subretinal adipose tissue-derived mesenchymal stem cell (ADMSC) implantation.	Ocular complications.
			Evidence of the short-term safety of ADMSCs in humans
Treatment of macular degeneration using embryonic stem cell-derived retinal pigment epithelium: preliminary results in Asian patients (NCT01625559 and NCT01674829) [13]	Dry age-related macular degeneration.	Subretinal transplantation of human embryonic-stem-cell (hESC)-derived retinal pigment epithelium.	No evidence of adverse serious safety issues.
	Stargardt macular dystrophy.		Visual acuity improvement.
Intravitreal autologous bone marrow CD34+ cell therapy for ischemic and degenerative retinal disorders: preliminary phase 1 clinical trial findings (NCT01736059) [12]	Irreversible vision loss from retinal vascular occlusion, hereditary or nonexudative age-related macular degeneration, or retinitis pigmentosa.	Intravitreal human bone marrow (BM) CD34+ stem cell injection.	No intraocular inflammation or hyperproliferation.
			Mild progression of geographic atrophy in AMD patients.
A phase I clinical trial of human embryonic stem cell-derived retinal pigment epithelial cells for early-stage Stargardt macular degeneration: 5-years’ follow-up (NCT02749734) [19]	Early-stage of Stargardt macular degeneration (STGD1).	Subretinal transplantation of human embryonic stem cell-derived retinal pigment epithelial (hESC-RPE) cells.	Safe and tolerable. Increased visual function.
			Visual function loss in two patients.
Long-term safety of human retinal progenitor cell transplantation in retinitis pigmentosa patients (ChiCTR-TNRC-08000193) [18]	Advanced retinitis pigmentosa.	Transplantation of purified human fetal-derived retinal progenitor cells (RPCs)	No immunological rejection or tumorigenesis. Long-term safety and feasibility.
			Significant improvement in visual acuity and increase in retinal sensitivity.

## Data Availability

Data used to write this review were taken from the public database ClinicalTrials.gov (https://clinicaltrials.gov/ (accessed on 10 September 2021)) and PubMed.gov (https://pubmed.ncbi.nlm.nih.gov/ (accessed on 10 September 2021)). All data supporting reported results can be found using the search terms published in this article.

## Supplementary Materials

The following are available online at https://www.mdpi.com/article/10.3390/medicina58010102/s1.
